# miR-27b-3p aggravates steatohepatitis progression by targeting the Nrf2/HO-1 pathway

**DOI:** 10.5713/ab.260476

**Published:** 2026-07-27

**Authors:** Kui Wang, Hao Pan, Yan Tian, Kangfeng Jiang, Xiaobing Li

**Affiliations:** 1 College of Veterinary Medicine, Yunnan Agricultural University, Kunming, China

**Keywords:** miR-27b-3p, Nrf2/HO-1, Oxidative Stress, Steatohepatitis

## Abstract

**Objective:**

The current study aimed to investigate whether miR-27b-3p regulates oxidative stress and inflammatory responses through the Nrf2/HO-1 signaling pathway during the progression of fatty liver. Liver tissues from dairy cows suffering from fatty liver and high-fat diet (HFD)-induced fatty liver mice were gathered to assess lipid accumulation, oxidative stress, inflammation, and the expression of miR-27b-3p and components of the Nrf2/HO-1 pathway.

**Methods:**

Liver tissues from dairy cows with fatty liver and HFD-induced fatty liver mice were collected to evaluate lipid accumulation, oxidative stress, inflammation, and the expression of miR-27b-3p and components of the Nrf2/HO-1 pathway. An *in vitro* steatosis model was established in AML-12 hepatocytes using palmitic acid (PA). Oxidative stress markers (reactive oxygen species [ROS], superoxide dismutase [SOD], and malondialdehyde [MDA]), inflammatory cytokines (IL-1β, IL-6, and TNF-α), and proteins associated with apoptosis were assessed. Bioinformatic analysis and dual-luciferase reporter assays were used to determine the interaction between miR-27b-3p and Nrf2. Functional assays were performed using miR-27b-3p mimetics and antagonists to evaluate its regulatory effects on the Nrf2/HO-1 signaling pathway and downstream cellular responses.

**Results:**

Fatty liver tissues from dairy cows and HFD-fed mice exhibited significant lipid deposition, enhanced oxidative stress, and elevated inflammatory responses, accompanied by upregulation of miR-27b-3p expression and reduced Nrf2/HO-1 signaling activity. Comparable alterations were observed in PA-treated AML-12 cells. Bioinformatic analysis and dual-luciferase experiments validated that miR-27b-3p directly binds to the 3′ untranslated region of Nrf2. Inhibition of miR-27b-3p restored Nrf2 and HO-1 expression, reduced ROS and MDA levels, enhanced SOD activity, suppressed NF-κB activation, decreased pro-inflammatory cytokine production, and alleviated apoptosis. Conversely, the overexpression of miR-27b-3p further inhibited Nrf2/HO-1 signaling and exacerbated oxidative stress, inflammation, and cellular damage.

**Conclusion:**

miR-27b-3p accelerates the progression of fatty liver toward steatohepatitis by directly inhibiting Nrf2 and downregulating the Nrf2/HO-1 antioxidant pathway, which increase oxidative stress, inflammatory responses, and hepatocyte apoptosis. These findings identify miR-27b-3p as a key upstream regulator of redox imbalance in fatty liver and suggest that targeting miR-27b-3p could be a promising strategy for the prevention and treatment of bovine fatty liver disease.

## INTRODUCTION

Fatty liver disease (FLD) is one of the most prevalent metabolic disorders affecting high-producing dairy cows during the transition period. Negative energy balance (NEB) after calving promotes excessive mobilization of non-esterified fatty acids (NEFAs) from adipose tissue to the liver, resulting in triglyceride (TG) accumulation and hepatic steatosis [1]. In severe cases, fatty liver may progress to steatohepatitis, characterized by oxidative stress, inflammation, hepatocyte injury, and impaired liver function, leading to substantial economic losses in the dairy industry [2].

Oxidative stress is considered a key driver of fatty liver progression [3]. Excessive lipid accumulation promotes reactive oxygen species (ROS) generation, disrupts redox homeostasis, and triggers inflammatory responses and hepatocyte apoptosis [4]. Nuclear factor erythroid 2-related factor 2 (Nrf2) is a master regulator of cellular antioxidant defense. Upon activation, Nrf2 translocates into the nucleus and induces the expression of antioxidant genes, including heme oxygenase-1 (HO-1), thereby protecting cells from oxidative damage [5]. Impairment of the Nrf2/HO-1 pathway has been associated with the development and progression of metabolic liver diseases.

MicroRNAs (miRNAs) are small non-coding RNAs that regulate gene expression at the post-transcriptional level and participate in diverse biological processes, including lipid metabolism, oxidative stress, and inflammation [6]. Emerging evidence suggests that miRNAs play a significant role in liver metabolic diseases [7]. In ruminants, it has been reported that liver-related miRNAs are involved in metabolic adaptation under energy-deficient conditions, further highlighting their regulatory role in liver metabolism [8]. Among them, miR-27b-3p has been implicated in metabolic regulation and inflammatory responses [9]. Moreover, bovine miRNAs have been shown to regulate adipocyte differentiation through AMPK/mTOR signaling, emphasizing the importance of miRNA-mediated regulation in lipid metabolism [10]. However, its role in dairy cow fatty liver and the underlying molecular mechanisms remain poorly understood.

Therefore, this study investigated the involvement of miR-27b-3p in fatty liver progression and explored its regulatory relationship with the Nrf2/HO-1 signaling pathway. Using liver tissues from dairy cows and high-fat diet (HFD)-induced mice, together with a palmitic acid-treated hepatocyte model, we examined whether miR-27b-3p modulates oxidative stress, inflammation, and apoptosis through targeting Nrf2. Our findings provide new insights into the molecular mechanisms underlying fatty liver progression and identify miR-27b-3p as a potential therapeutic target for FLD.

## MATERIALS AND METHODS

### Animals experiment

The dairy cows used in this study were selected from a commercial dairy farm in Xundian, Yunnan Province, China. Prior to experimentation, all animals underwent routine physical examinations to confirm disease-free status. During the sampling period, *ad libitum* feeding was maintained with identical dietary rations across the herd. Liver biopsy and sampling were performed by licensed veterinarians using aseptic techniques. By measuring the TG content in the livers of cows within two weeks after giving birth, they were categorized into severe, moderate, and healthy groups (with six cows in each group).

Twenty male Kunming mice aged 6–8 week were procured from the Experimental Animal Center of Kunming Medical University. All housing facilities underwent thorough sterilization of cages, water bottles, and environmental surfaces prior to experimentation and the mice were fed in a Specific Pathogen-Free (SPF) environment. After a 1-week acclimatization period under standard laboratory conditions, mice were stratified by body weight into two groups (n = 10/group): control diet (CD) and HFD groups. The HFD group received XTMCD-formulated feed (Xietong Biotech), while the CD group maintained standard chow. Both groups were fed continuously for 8 weeks and had *ad libitum* access to food and water throughout the 8-week intervention period, hepatic tissues were harvested terminally.

### Histopathological analysis

Histopathological staining was performed as previously described [11]. Briefly, the collected tissues were fixed overnight in 4% paraformaldehyde, embedded in paraffin, sectioned and stained. Histopathological changes in the liver tissues were observed under an optical microscope (Olympus).

### Reactive oxygen species assaying of liver tissue

Liver tissue samples (5 mm^3^) were immediately placed in pre-chilled 1.5 mL cryovials, flash-frozen in liquid nitrogen, and transported to Servicebio for ROS fluorescence staining using dihydroethidium (DHE) fluorescent probes.

### Intracellular reactive oxygen species quantification

After treatment, the DCFH-DA fluorescent probe (abs42197174; abinScience) is pre-diluted with serum-free medium at a ratio of 1:500, and prepared at a concentration of 500 μL per well in a 12-well plate. Add the probe and incubate in a 37°C incubator for 20 minutes. After incubation, rinse three times with serum-free medium, incubate at room temperature with Hoechst (C1025; Beyotime) for 5–10 minutes for cell nucleus staining, and observe under a fluorescence microscope.

### Evaluation of liver biochemical function

According to the instructions of the Nanjing Jiancheng Biochemical Kit, Serum TG (Cat#A110-1-1), total cholesterol TCHO (Cat#A111-1-1); alanine aminotransferase (ALT) (Cat#C009-2-1), aspartate transaminase (AST) (Cat#C010-2-1) was measured. According to the manufacturing instructions of the reagent kit, superoxide dismutase (SOD, Cat#BC6415) (Solarbio) and malondialdehyde (MDA, Cat#BC5165) (Solarbio) was measured.

### Cell culture and viability analysis

The Alpha Mouse Liver 12 (AML-12) cell line was purchased from the cell bank of the Chinese Academy of Sciences and cultured and cryopreserved in our laboratory. Cultivate in Dulbecco’s Modified Eagle Medium (DMEM/F12, Thermo Fisher Scientific) containing 10% fetal bovine serum (10–6 mol/L insulin, 10–6 mol/L dexamethasone) (cell box; Hunan) and 1% triple antibiotics (Thermo Fisher Scientific), 37°C, 5% CO_2_. HEK 293T cells were donated by the School of Veterinary Medicine at Huazhong Agricultural University and preserved in our laboratory. Cultivate in DMEM (Thermo Fisher Scientific) containing 10% fetal bovine serum (cell box; Hunan) and 1% triple antibiotics (Thermo Fisher Scientific), 37°C, 5% CO_2_. The cytotoxicity of different concentrations of PA (0, 200, 400, 600, 800, 1,000, 1,200 μM) on AML-12 cells for 24 hours was detected using Cell Counting Kit-8 (CCK-8; Cat#BS350A; Bioss). The absorbance at 450 nm of each well was detected by microplate reader (Feyond-A300) and the data were calculated.

### TUNEL apoptosis assay

After treatment, cells were fixed with 4% paraformaldehyde, and apoptosis was assessed using a TUNEL staining kit according to the manufacturer’s instructions.

### Western blotting

Total protein was extracted from tissues or cells using a commercial kit (Solarbio). Western blotting was performed as previously described [12]. In brief, Bio-Rad electrophoresis transfer device and PVDF membrane were used for protein transfer and membrane transfer using SDS/PAGE mixture for electrophoresis. The membrane was subsequently placed in commercial rapid blocking solution (Shanghai Yazyme) for 15 min, after which the primary antibodies were incubated at 4°C overnight. The following primary antibodies were used: NF-κB p65 (CST, #8242S), P NF-κB p65 (AP1294; Abclonal), β-Actin (CST, #4970), Nrf2 (CST, #20733T), Bax (Ab32503; Abcam), Bcl-2 (CST, #28150), HO-1 (bs-23397R; Bioss), Anti-rabbit (CST, #7074S). β-actin was used as an internal reference protein to calculate the expression of target protein

### Quantitative reverse transcription polymerase chain reaction

The cells and tissue were lysed using RNAiso Plus (Takara). After reverse transcription, quantitative polymerase chain reaction (qPCR) analysis was performed using PerfectStart Green qPCR SuperMix (TRANS) on a real-time PCR system (Analytikjena). Primer sequences are provided in Tables 1–3.

### Electroporation transfection of cells

miR-27b-3p mimics, miR-27b-3p inhibitor and Nrf2 overexpression plasmid were synthesized by GenePharma. Transfection was performed according to the manufacturer’s protocol for electroporation machines (BTX Gemini SC, Cat. No. 452043).

### Flow cytometry

AML-12 cells exhibiting optimal growth characteristics were inoculated in six-well plates and treated with varying concentrations of PA (Cat#P5585; Sigma-Aldrich) dye for 24 hours. Following this, the cells were washed three times with PBS, digested using EDTA-free trypsin, and transferred to centrifuge tubes. The cells were resuspended by adding 1 mL of DCFH-DA (S0033S; Beyotime) dye working solution configured in serum-free medium to the cell precipitate and incubated at 37°C with light-protected conditions for 30 minutes. Thereafter, the cells were washed with a serum-free medium once, and the intracellular ROS level was detected by flow cytometry.

### Dual-luciferase reporter assay

By predicting the binding site between miR-27b and Nrf2 through the Targeted Scan bioinformatics website, PCR technology was used to amplify the partial sequence of the Nrf2 gene 3′UTR containing this binding site. The amplified product was transfected into pSI-Check2 to construct the Nrf2 wild-type (Nrf2 WT) vector. In addition, according to the instructions of the Vazyme Mut Express II Mutagenesis Kit V2 kit, Nrf2 mutant primers were designed. HEK 293T cells were seeded into a 6-well plate and co-transfected with either Nrf2 WT or Nrf2 MUT vectors, along with miR-27b mimics or negative controls. After 36–48 hours of transfection, luciferase activity was measured using the Dual-Luciferase Reporter Assay System (Cat# 11402ES60; Yeasen Biotechnology) with firefly luciferase as internal control.

### Statistical analysis

Data are presented as mean±SEM. Statistical analyses were performed using GraphPad Prism 9.0. In the comparison between the two independent groups, the unpaired, two-tailed Student’s t-test was used. One-way analysis of variance (ANOVA) followed by Tukey’s multiple comparisons test was used for comparisons among multiple groups. p-value less than 0.05 is considered statistically significant, and the marks in the figure are as follows: * p<0.05, ** p<0.01, *** p<0.001.

## RESULTS

### Biochemical identification and detection of oxidative stress and inflammation in dairy cow fatty liver

Compared with healthy cows, cows with fatty liver had higher levels of TG and TCHO in their liver, indicating significant lipid accumulation (Figure 1A, 1B). The increase in AST and ALT activity indicates impaired liver function (Figure 1C, 1D). Histological analysis further revealed vacuolization of liver cells and increased lipid deposition in fatty liver tissue (Figure 1E). To investigate the antioxidant status within the organization, SOD activity and MDA content in plasma were measured. The results showed that SOD activity in fatty liver tissue decreased, MDA content increased, and ROS fluorescence intensity increased (Figure 1F–1H). The reverse transcription quantitative polymerase chain reaction (RT-qPCR) results indicated a significant decrease in the expression of the Nrf2 gene and a significant increase in the expression of miR-27b in the fatty liver model (FL) group (Figure 1I, 1J). Western blot analysis (Figure 1K) confirmed that both Nrf2 and its downstream target HO-1 protein levels were significantly reduced in the FL group compared to healthy cows. Inflammatory level was assessed by RT-qPCR. The mRNA levels of IL-1β, IL-6, and TNF-α were significantly higher in the FL group compared to the healthy group (Figure 1L–1N). Furthermore, the phosphorylation of NF-κB p65 was markedly increased in the FL group (Figure 1O). These results indicate that bovine fatty liver is accompanied by lipid accumulation, oxidative stress, and inflammatory activation.

### Biochemical identification and detection of oxidative stress and inflammation in mice fatty liver model

Ten mice were used to induce a FL, with another set of 10 mice serving as controls. After feeding the HFD for 8 weeks, liver dissection was performed on both groups. Similar alterations in serum biochemical parameters were observed in HFD-fed mice, including increased TG, TCHO, AST, and ALT levels (Figure 2A–2D). H&E staining revealed vacuolar steatosis in the liver cells of fatty liver mice, while Oil Red O staining indicated severe lipid accumulation (Figure 2E). These results confirm the successful establishment of the FL in mice.

To investigate whether oxidative stress occurs in the liver of fatty liver mice, we measured SOD and MDA levels, along with the intensity and distribution of ROS. The results indicated that HFD feeding decreased SOD activity and increased MDA content and ROS fluorescence intensity (Figure 2F–2H). In addition, the mRNA expression level of Nrf2 was significantly decreased in fatty liver mice (Figure 2I). On the contrary, the gene expression level of miR-27b-3p was significantly increased (Figure 2J). The results of protein expression of Nrf2 and HO-1 further showed that the oxidative stress state of liver was active (Figure 2K). Furthermore, the expression levels of inflammatory markers IL-1β, IL-6, TNF-α were significantly upregulated in fatty liver mice (Figure 2L–2N). Western blotting analysis showed activation of the NF-κB pathway, which confirmed that there was severe inflammation in the liver of fatty liver mice (Figure 2O). These findings are consistent with the changes observed in cows.

### Oxidative stress and inflammatory response induced by palmitic acid in AML-12

To construct the *in vitro* model of fatty liver, we treated AML-12 cells with PA concentrations ranging from 0–200–400–600–800–1,000–1,200 μM. According to CCK-8 assay, AML-12 cell viability decreases with increasing PA concentration. At 800 μM PA, cells still maintain good viability (>85), while higher concentrations (>800 μM) can lead to excessive cytotoxicity (Figure 3A). In addition, intracellular TG accumulation significantly increased at 800 μM PA (Figure 3B). Therefore, 800 μM PA was chosen as the optimal concentration because it induced lipid accumulation and maintained sufficient cell viability for subsequent mechanism studies. To evaluate the oxidative stress promoting effect of PA *in vitro*, we measured the levels of SOD and MDA in AML-12 cells after PA treated. The results showed that PA treatment decreased SOD activity and increased MDA content and ROS production (Figure 3C–3E). This indicates that AML-12 cells experienced oxidative stress after PA stimulation, and ROS levels significantly increased. The qPCR detection results of miR-27b-3p and Nrf2 gene expression showed that the expression of miR-27b-3p gene was significantly increased (Figure 3F) while Nrf2 mRNA level was significantly decreased (Figure 3G) in the *in vitro* model of fatty liver. The Western blot results showed that the expression of Nrf2 protein was significantly reduced, and the expression of HO-1 protein was significantly reduced (Figure 3H), indicating that PA inhibited the Nrf2/HO-1 antioxidant pathway. This further indicates the oxidative stress caused by PA in AML-12 cells. Moreover, PA increased IL-1β, IL-6, and TNF-α expression, enhanced NF-κB p65 phosphorylation, and altered apoptosis-related protein expression, as shown by decreased Bcl-2 and increased Bax expression (Figure 3I–3L). These results indicate that PA induced oxidative stress, inflammatory activation, and hepatocyte injury in AML-12 cells.

### Biological prediction of miR-27b-3p and Nrf2 genes

The results above demonstrate that miR-27b-3p is upregulated in the FL, while Nrf2 expression follows an opposite trend. This suggests that miR-27b may target Nrf2 to regulate its activity. Bioinformatic analysis predicted a conserved binding site for miR-27b-3p in the 3′ UTR of Nrf2 (Figure 4A). To verify this interaction, wild-type and mutant Nrf2 3′ UTR luciferase reporter vectors were constructed. miR-27b-3p mimic significantly reduced the luciferase activity of the wild-type Nrf2 3′ UTR reporter, whereas no significant change was observed in the mutant reporter group (Figure 4B). These results confirm that miR-27b-3p directly targets the 3′ UTR of Nrf2.

### Inhibiting mir-27b-3p alleviates fatty liver damage

To determine the role of miR-27b-3p in PA-induced hepatocyte injury, AML-12 cells were transfected with a miR-27b-3p inhibitor. Compared with the NC + PA group, miR-27b-3p inhibition increased SOD activity and reduced MDA content and ROS production (Figure 5A–5C). Inhibition of miR-27b-3p also restored Nrf2 and HO-1 protein expression (Figure 5D). Furthermore, miR-27b-3p inhibition decreased the expression of IL-1β, IL-6, and TNF-α and reduced NF-κB p65 phosphorylation (Figure 5E–5H). Apoptosis-related changes were also alleviated, as indicated by improvement in the Bcl-2/Bax ratio (Figure 5H). These data suggest that miR-27b-3p inhibition protects AML-12 cells against PA-induced oxidative and inflammatory injury.

### Overexpression of mir-27b-3p enhances fatty liver damage

To further investigate the effect of miR-27b-3p on the progression of fatty liver, AML-12 cells were transfected with miR-27b-3p mimic. Compared with the NC+PA group, miR-27b-3p overexpression further decreased SOD activity and increased MDA content and ROS production (Figure 6A–6C). Nrf2 and HO-1 protein expression was further reduced in the mimic+PA group (Figure 6D). In addition, miR-27b-3p overexpression enhanced IL-1β, IL-6, and TNF-α expression, increased NF-κB p65 phosphorylation, and aggravated apoptosis-related protein changes (Figure 6E–6H). These results indicate that miR-27b-3p overexpression exacerbates PA-induced oxidative stress, inflammation, and hepatocyte injury.

### Nrf2 overexpression rescues miR-27b-3p-induced oxidative stress and inflammation

Nrf2 overexpression was performed by transfecting Nrf2 overexpression plasmids to investigate whether Nrf2 mediates the effects of miR-27b-3p in PA-treated AML-12 cells. Compared with the PA+miR-27b-3p simulation group, overexpression of Nrf2 restored Nrf2 and HO-1 protein levels, and reduced ROS accumulation (Figure 7A, 7B). In addition, NF-κB p65 activation was reduced and the accumulation of inflammatory factors was decreased (Figure 7C, 7D). These results indicate that the restoration of Nrf2 expression weakens miR-27b-3p mediated oxidative stress and inflammatory activation in PA treated liver cells.

## DISCUSSION

Nonalcoholic fatty liver disease (NAFLD) is a common metabolic disease in humans characterized by hepatic lipid accumulation and oxidative stress [13], which bears a striking resemblance to FLD in dairy cows. However, FLD in dairy cows exhibits unique characteristics arising from species-specific physiological needs. FLD often occurs in dairy cows during the perinatal period, which leads to the decline of milk production and reproductive performance [3]. As a root cause, the long-term NEB triggers fat compensation in the dairy cow’s body, which further produces a large amount of NEFA, causing inflammatory damage and oxidative stress to liver cells [14]. These NEFAs are subsequently re-esterified into TG in the liver. Similarly, we observed that cow fatty liver suffered from severe oxidative stress and inflammation. Burke et al. [15] reported elevated activities of key liver function markers, AST and ALT, in transitional dairy cows, suggesting hepatic damage during the periparturient period. In this study, biochemical analysis of hepatic tissue revealed significantly increased AST and ALT activities, further confirming impaired liver function in dairy cows with fatty liver. The liver, as an energy transfer organ, becomes overloaded with NEFA. On the one hand, inflammation induces the production of mitochondrial ROS through the respiratory burst of macrophages [16], on the other hand, oxidative stress activates NF - κ B pathway and promotes the production of more ROS [17], which makes this vicious cycle more vicious. Research reports have shown that genetic manipulation of metabolic regulators such as Vanin-1 significantly affects liver lipid metabolism and oxidative status, further supporting the importance of redox-related pathways in liver metabolic homeostasis [18]. Consistent with our observations, redox imbalance in perinatal dairy cows is closely associated with enhanced lipid mobilization and oxidative stress. While inflammation is essential for host defense, chronic or excessive inflammation can cause significant tissue damage.

Infiltration of inflammatory cells is a hallmark of steatohepatitis progression, which actively promotes disease progression. Previous study has shown that high concentrations of NEFA can cause lipid metabolism disorders with severe inflammation [19]. Loor et al. [20] reported that a positive correlation between NEFA concentration and liver inflammation in early lactation. In this study, NF-κB signal was activated in moderate to severe fatty liver of dairy cattle, and the expression of inflammatory cytokines (TNF-α, IL-1 β and IL-6) in liver tissue was up-regulated. Oxidative stress and inflammation are two interrelated pathological processes in the development of fatty liver. Trevisi and Minuti demonstrated a positive correlation between oxidative stress and inflammation in transition dairy cows [21]. We have advanced this view in the liver tissue of mice fed with HFD.

PA-induced steatosis models are widely used to investigate the molecular mechanisms underlying lipid accumulation and hepatocyte injury [22]. Alnahdi et al. [23] reported that PA treatment markedly elevated ROS levels, decreased the GSH/GSSG ratio, inhibited SOD activity, and enhanced the release of inflammatory cytokines. Fang et al. [24] observed that PA induced lipid metabolic disturbance, endoplasmic reticulum stress, and inflammation in primary fish intestinal cells. In the present study, PA stimulation of AML-12 cells successfully established an *in vitro* lipid accumulation injury model, further confirming that PA induces oxidative stress and activates inflammatory responses in hepatocytes. A recent study elucidated how PA promotes oxidative stress and NASH progression, demonstrating that peroxiredoxin 1 (Prdx1) activity can effectively mitigate NASH [25]. These findings underscore the critical role of antioxidants in reversing fatty liver progression.

Many pieces of evidence indicate that miR-27b-3p is involved in lipid metabolism, oxidative stress, and inflammatory responses in different species [26,27]. Clinical studies report a strong correlation between serum miR-27 levels and disease severity in NAFLD patients [28,29]. Previously, we analyzed significant protein binding term and miRNA pathway enrichment in samples of bovine steatohepatitis (Supplements 1, 2). Collectively, these findings underscore miR-27b as a multifunctional regulator of lipid homeostasis and oxidative stress, yet its role in bovine NAFLD remains unexplored. We aimed to investigate the effect of miR-27b-3p on fatty liver damage.

miRNA can binds to the 3′ UTR end of the target gene, inhibits gene transcription through incomplete complementary binding, or mediates target gene degradation through complete complementary binding, thereby regulating target gene expression and subsequent protein synthesis [30]. At present, there are many miRNAs that regulate oxidative stress and inflammatory damage through target genes. A study reported that miR-200a regulates Nrf2 antioxidant pathway by targeting Keap1 to prevent fructose induced liver inflammation and oxidative stress [31]. In addition, activation of the NRF2-ARE signaling pathway has been demonstrated to alleviate oxidative stress and improve cellular function in animal models, further supporting the protective role of NRF2 signaling observed in the present study [32]. Min Gao found that miR-1-3p regulates inflammation and oxidative stress of endothelial cells by regulating the expression of target gene serp1 [33]. While previous studies have implicated miR-27a in lipid metabolism by targeting PPARγ [34], our findings highlight a distinct role of miR-27b-3p in redox regulation. Contrast with miR-27a, which primarily modulates adipogenesis, miR-27b-3p exacerbates NAFLD progression through direct inhibition of Nrf2, suggesting functional divergence within the miR-27 family. Previous research reports have stated that Geniposide (GEN) slows down H2O2 induced oxidative stress and restores Nrf2 expression in human liver cell oxidative damage by targeting miR-27b-3p [9]. However, it is unclear whether the miR-27b-3p/Nrf2 pathway is involved in the progression of fatty liver in dairy cows. This study demonstrates that miR-27b-3p is upregulated in fatty liver tissue from cattle, suggesting its potential role in the disease. The *in vitro* data further supports this viewpoint. Our findings suggest that the miR-27b-3p/Nrf2 axis regulates oxidative stress and inflammatory damage during the progression of bovine fatty liver. The RT-qPCR results revealed a significant upregulation of miR-27b-3p and downregulation of Nrf2 in both bovine and murine FL. Dual-luciferase reporter assays confirmed that Nrf2 is a direct target of miR-27b-3p. Furthermore, its regulatory role in the progression of steatohepatitis was clarified by mimics and inhibitors. Collectively, these findings establish miR-27b-3p as an upstream regulator that links lipid overload to Nrf2 suppression in bovine steatohepatitis.

The Nrf2 overexpression rescue experiment further strengthened the mechanistic interpretation of this study. Although previous reports have described the interaction between miR-27b-3p and Nrf2, our rescue data indicate that restoration of Nrf2 partially reverses miR-27b-3p-induced lipid accumulation and oxidative injury in the *in vitro* steatosis model. Therefore, the present findings do not simply identify Nrf2 as a target of miR-27b-3p, but provide functional evidence supporting the involvement of Nrf2 in miR-27b-3p-mediated hepatic lipid metabolic disorder and oxidative stress.

In order to clarify the role of miR-27b-3p in cross species steatohepatitis, we used a mouse liver cell line to induce steatohepatitis, but it could not be completely used in clinical cow steatohepatitis. Future studies would benefit from utilizing primary bovine liver cells or organoid models. Our research findings provide supporting evidence for the biological function of miR-27b-3p/Nrf2 axis in lipid accumulation induced oxidative stress and inflammation in perinatal dairy cows. However, Nrf2 is a classical antioxidant stress-responsive transcription factor that regulates multiple downstream antioxidant targets in addition to HO-1. Therefore, future research will benefit from the confirmation of more downstream Nrf2 targets.

## CONCLUSION

In summary, our results indicate that miR-27b-3p inhibits antioxidant stress in fatty liver and triggers liver cell inflammatory damage by targeting Nrf2 during the progression of non-alcoholic steatohepatitis in cows and mice. These findings highlight the key role of miR-27b-3p in the pathogenesis of bovine fatty liver oxidative stress and inflammatory damage and demonstrate its potential as a therapeutic target and rapid biomarker for detection (Figure 8).

## Figures and Tables

**Figure 1:**
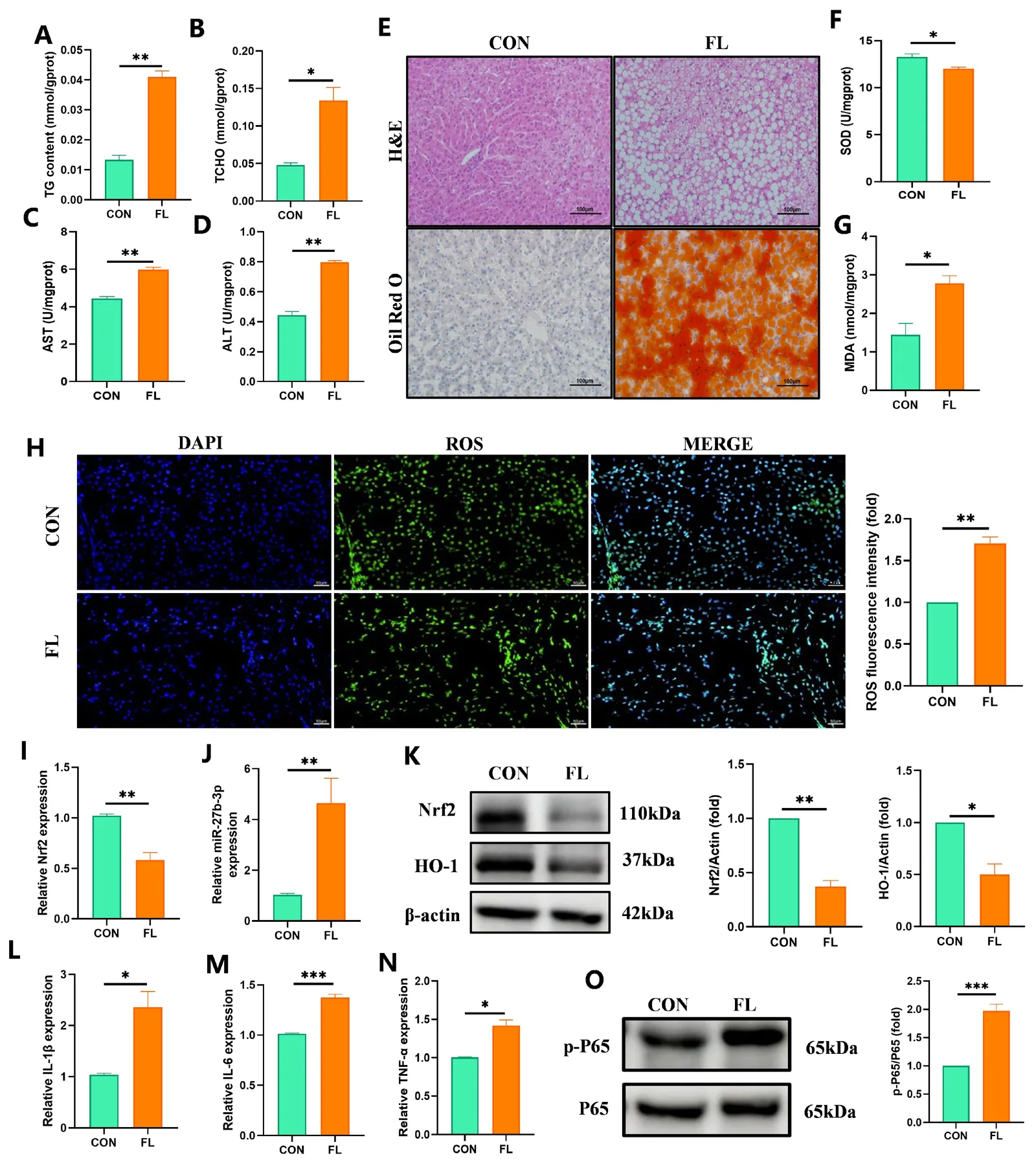
Biochemical identification and detection of oxidative stress and inflammation in dairy cow fatty liver. (A) TG content in cows liver; (B) T-CHO content in cows liver; (C) The activity of AST; (D) The activity of ALT; (E) H&E and oil red O staining of the cows liver (200×); (F) The activity of SOD in cows liver; (G) The content of MDA in cows liver; (H) Fluorescence microscopy for ROS detection in cows liver (200×); (I): RT-qPCR detection of Nrf2 gene expression levels in cows liver; (J) RT-qPCR detection of miR-27b gene expression levels in cows liver; (K) Western blotting detection of Nrf2 and HO-1 protein expression levels in cows liver; (L) RT-qPCR detection of IL-1β gene expression levels in cows liver; (M) RT-qPCR detection of IL-6 gene expression levels in cows liver; (N) RT-qPCR detection of TNF-α gene expression levels in cows liver; (O) Western blotting detection of NF-κB p65 protein activation levels in cows liver. Data are expressed as the means±SEM. * p<0.05, ** p<0.01, *** p<0.001. TG, triglyceride; AST, aspartate transaminase; ALT, alanine aminotransferase; FL, fatty liver model; SOD, superoxide dismutase; MDA, malondialdehyde; ROS, reactive oxygen species; RT-qPCR, reverse transcription quantitative polymerase chain reaction; SEM, standard error of the mean.

**Figure 2:**
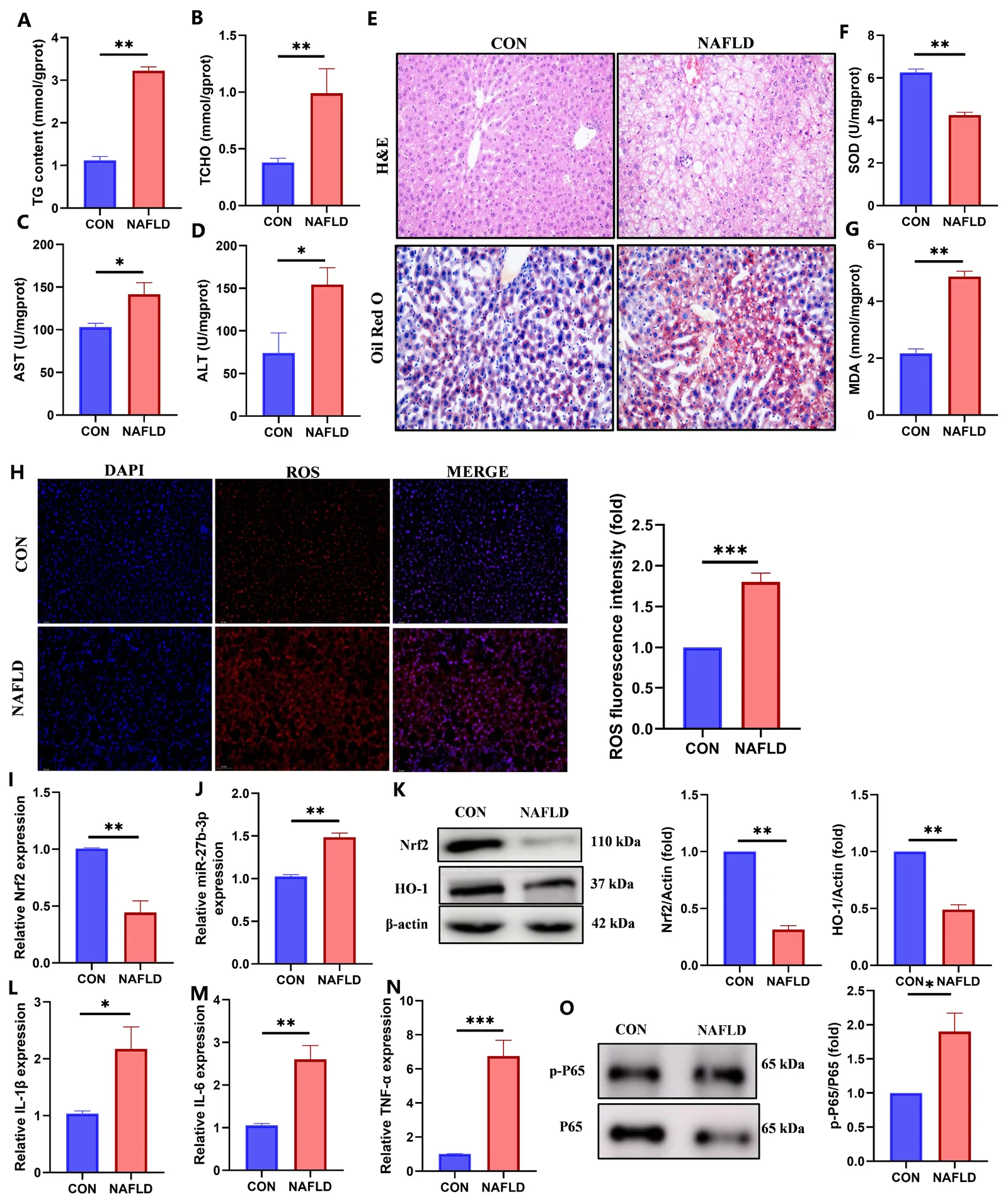
Biochemical identification and detection of oxidative stress and inflammation in mice fatty liver model. (A) TG content in mice liver; (B) T-CHO content in mice liver; (C) The activity of AST; (D) The activity of ALT; (E) H&E and oil red O staining of the mice liver (200×); (F) The activity of SOD in mice liver; (G) The content of MDA in mice liver; (H) Fluorescence microscopy for ROS detection in liver of mice (200×); (I) RT-qPCR detection of Nrf2 gene expression levels in liver of mice; (J) RT-qPCR detection of miR-27b gene expression levels in mice liver; (K) Western blotting detection of Nrf2 and HO-1 protein expression levels in mice liver; (L) RT-qPCR detection of IL-1β gene expression levels in mice liver; (M) RT-qPCR detection of IL-6 gene expression levels in mice liver; (N) RT-qPCR detection of TNF-α gene expression levels in mice liver; (O) Western blotting detection of NF-κB p65 protein activation levels in mice liver. Data are expressed as the means±SEM. * p<0.05, ** p<0.01, *** p<0.001. TG, triglyceride; AST, aspartate transaminase; ALT, alanine aminotransferase; NAFLD, nonalcoholic fatty liver disease; SOD, superoxide dismutase; MDA, malondialdehyde; ROS, reactive oxygen species; RT-qPCR, reverse transcription quantitative polymerase chain reaction; SEM, standard error of the mean.

**Figure 3:**
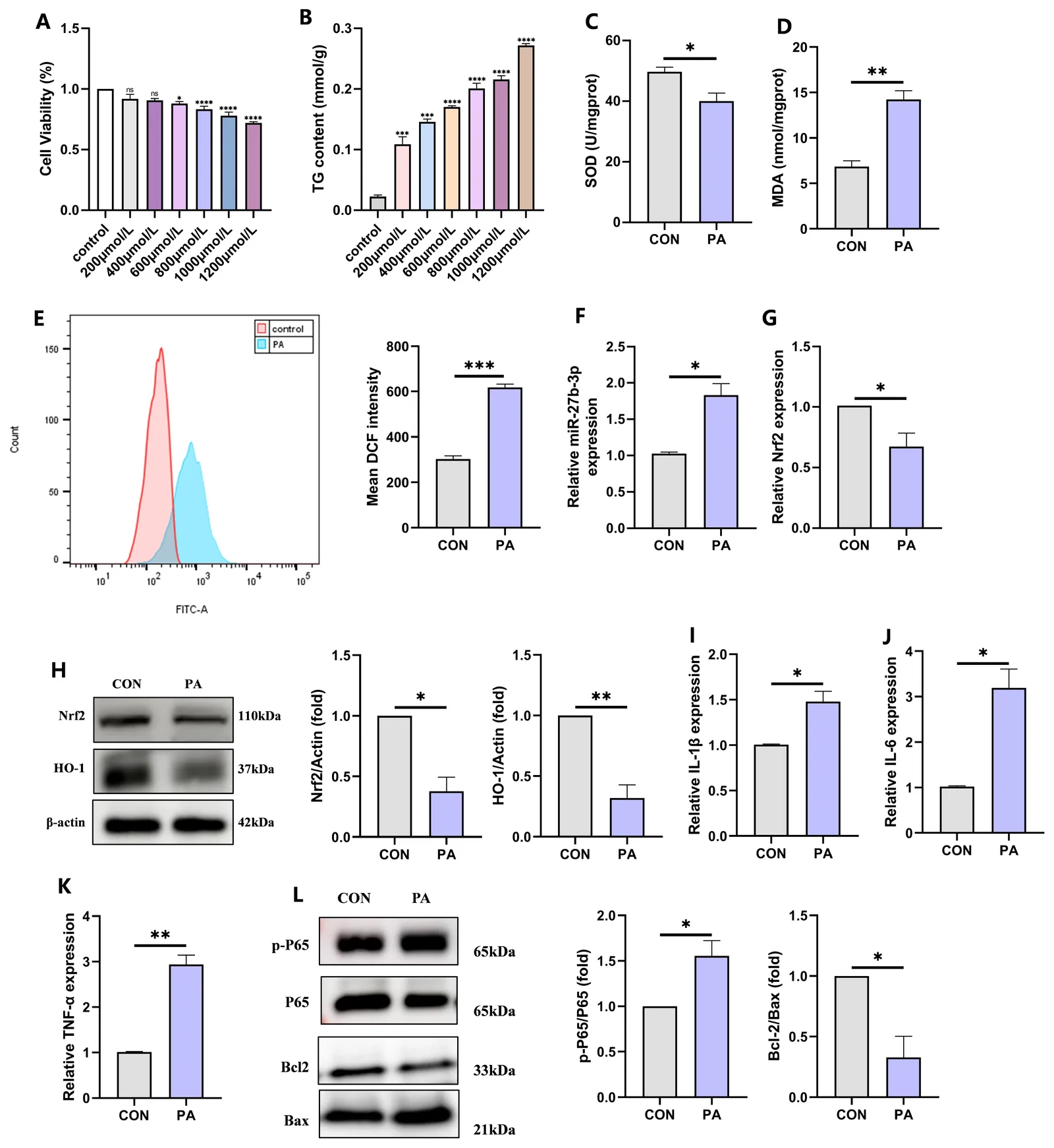
Oxidative stress and inflammatory response induced by PA in AML-12. (A) CCK-8 assay for cell viability; (B) TG content detection in AML-12; (C) The activity of SOD in AML-12; (D) The content of MDA in AML-12; (E) ROS levels in AML-12; (F) RT-qPCR detection of miR-27b gene expression levels in AML-12; (G) RT-qPCR detection of Nrf2 gene expression levels in AML-12; (H) Western blotting detection of Nrf2 and HO-1 protein expression levels in AML-12; (I) RT-qPCR detection of IL-1β gene expression levels in AML-12; (J) RT-qPCR detection of IL-6 gene expression levels in AML-12; (K) RT-qPCR detection of TNF-α gene expression levels in AML-12; (L) Western blotting detection of p-NF-κB p65 and Bcl2/Bax in AML-12. Data are expressed as the means±SEM. * p<0.05, ** p<0.01, *** p<0.001, **** p<0.0001. TG, triglyceride; SOD, superoxide dismutase; MDA, malondialdehyde; PA, palmitic acid; ROS, reactive oxygen species; RT-qPCR, reverse transcription quantitative polymerase chain reaction; SEM, standard error of the mean.

**Figure 4:**
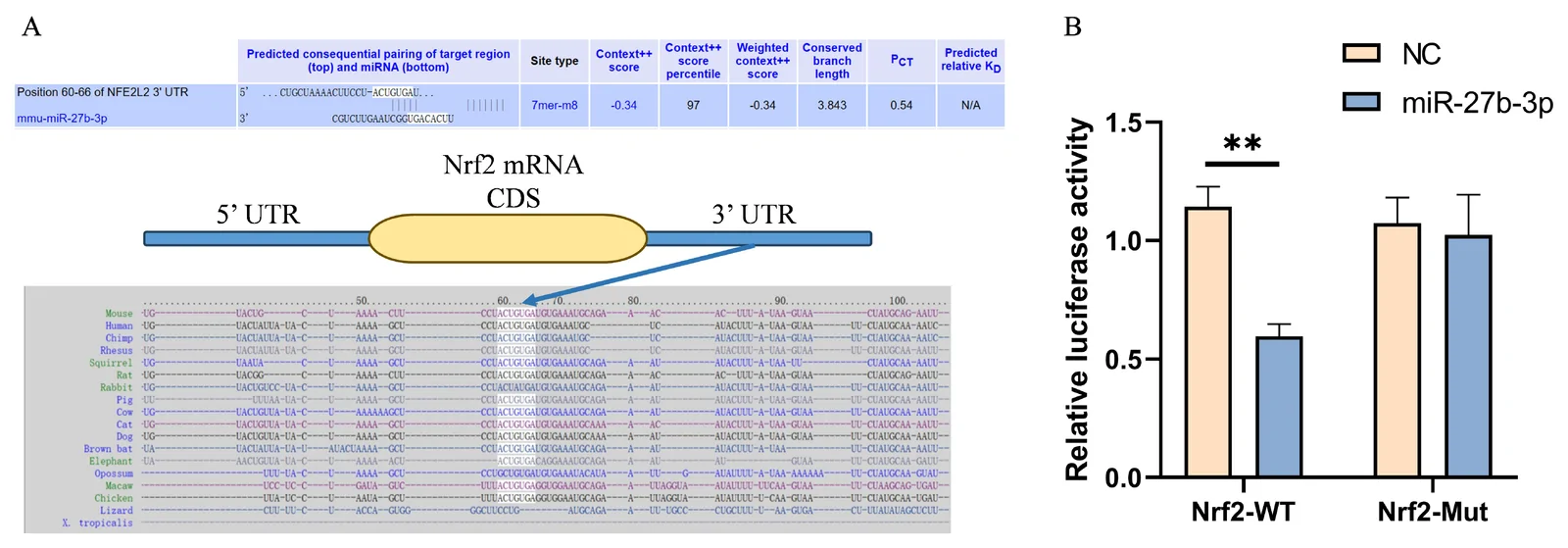
Biological prediction of miR-27b-3p and Nrf2 genes. (A) Prediction of miR-27b-3p binding site with Nrf2 gene 3′UTR; (B) Dual luciferase gene reporter data are expressed as the means±SEM. ** p<0.01. SEM, standard error of the mean.

**Figure 5:**
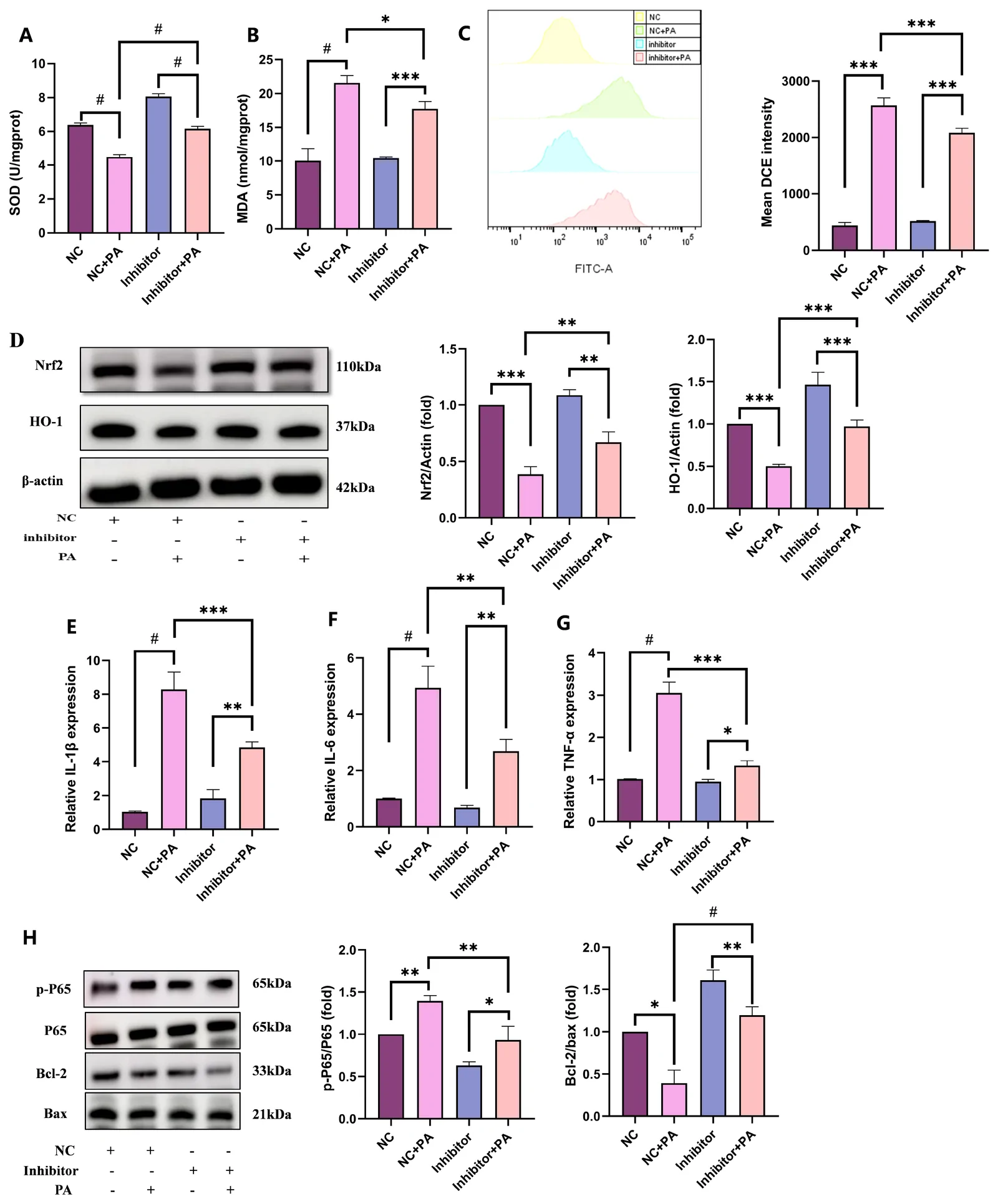
Inhibiting miR-27b-3p alleviates fatty liver damage. (A) The activity of SOD in AML-12; (B) The content of MDA in AML-12; (C) Detection of ROS levels in AML-12 by flow cytometry; (D) Western blotting detection of Nrf2 and HO-1 proteins expression levels in AML-12; (E) RT-qPCR detection of IL-1β gene expression levels in AML-12; (F) RT-qPCR detection of IL-6 gene expression levels in AML-12; (G) RT-qPCR detection of TNF-α gene expression levels in AML-12; (H) Western blotting detection of p-NF-κB p65 and apoptotic protein expression in AML-12. Statistical significance was assessed using one-way ANOVA; * p<0.05, ** p<0.01, *** p<0.001, ^#^ p<0.0001. SOD, superoxide dismutase; MDA, malondialdehyde; ROS, reactive oxygen species; RT-qPCR, reverse transcription quantitative polymerase chain reaction; ANOVA, analysis of variance.

**Figure 6:**
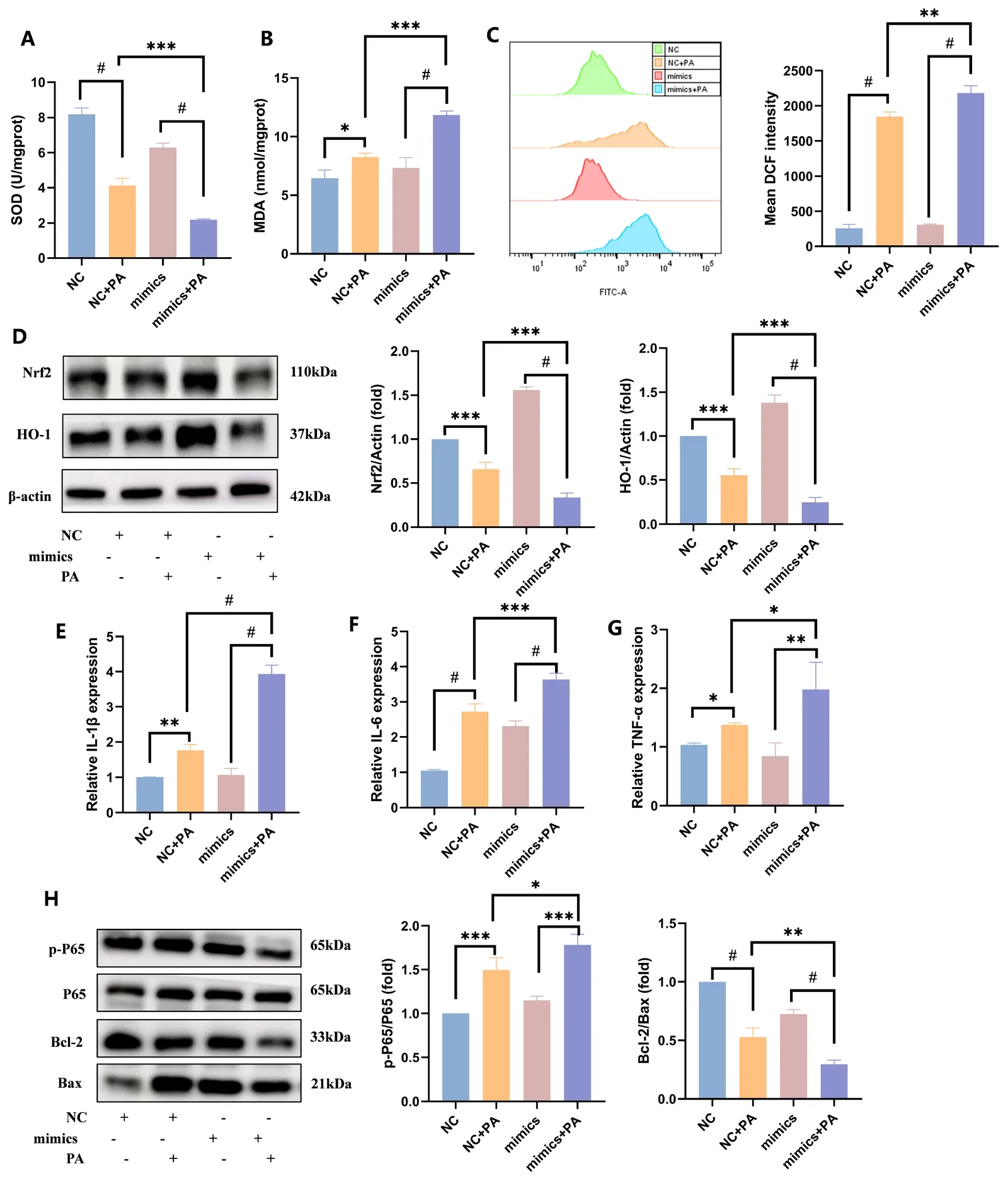
Overexpression of mir-27b-3p enhances fatty liver damage. (A) The activity of SOD in AML-12; (B) The content of MDA in AML-12; (C) Detection of ROS levels in AML-12 by flow cytometry; (D) Western blotting detection of Nrf2 and HO-1 proteins expression levels in AML-12; (E) RT-qPCR detection of IL-1β gene expression levels in AML-12; (F) RT-qPCR detection of IL-6 gene expression levels in AML-12; (G) RT-qPCR detection of TNF-α gene expression levels in AML-12; (H) Western blotting detection of p-NF-κB p65 and apoptotic protein expression in AML-12. Statistical significance was assessed using one-way ANOVA; * p<0.05, ** p<0.01, *** p<0.001, ^#^ p<0.0001. SOD, superoxide dismutase; MDA, malondialdehyde; ROS, reactive oxygen species; RT-qPCR, reverse transcription quantitative polymerase chain reaction; ANOVA, analysis of variance.

**Figure 7:**
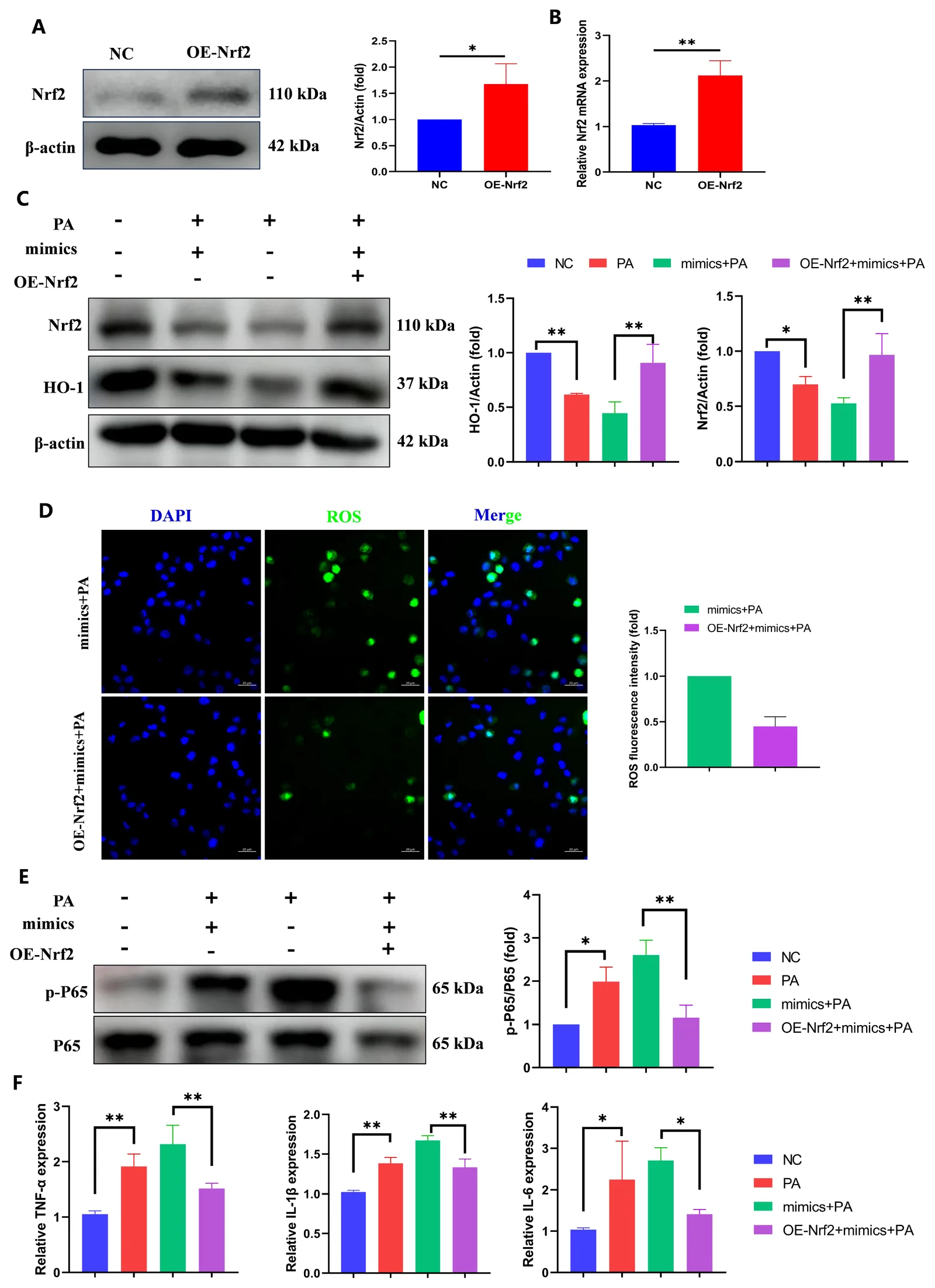
Nrf2 overexpression rescues miR-27b-3p-induced oxidative stress and inflammation. (A) Western blotting detection of Nrf2 protein expression in AML-12 cells transfected with Nrf2 overexpression plasmid. (B) RT-qPCR detection of Nrf2 mRNA expression in AML-12 cells after Nrf2 overexpression. (C) Western blotting detection of Nrf2 and HO-1 protein expression levels in AML-12 cells treated with PA, miR-27b-3p mimics, and Nrf2 overexpression plasmid. (D) Fluorescence microscopy detection of intracellular ROS levels in AML-12 cells. DAPI was used for nuclear staining, and green fluorescence indicates ROS production (400 x). (E) Western blotting detection of p NF-κB p65 and total NF-κB p65 protein expression levels in AML-12 cells. (F) RT-qPCR detection of TNF-α, IL-1β, and IL-6 mRNA expression levels in AML-12 cells. Data are expressed as the means±SEM. * p<0.05, ** p<0.01. PA, palmitic acid; ROS, reactive oxygen species; RT-qPCR, reverse transcription quantitative polymerase chain reaction; SEM, standard error of the mean.

**Figure 8:**
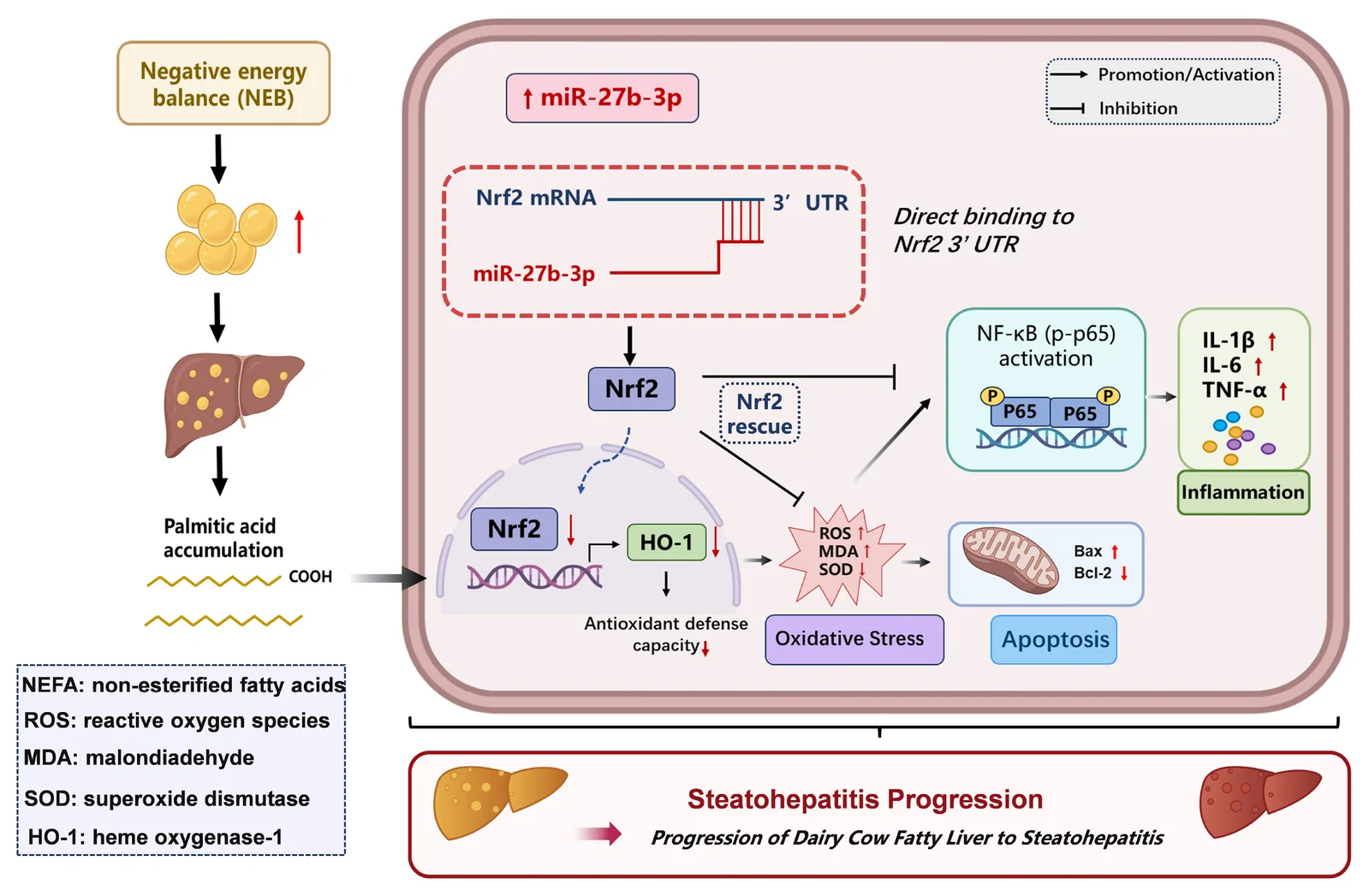
Schematic diagram of the molecular mechanism by which miR-27b-3p regulates hepatic steatosis. The figure was created with BioRender (BioRender.com) using a paid academic account.

**Table 1:** The primer sequences (5′–3′) of bovine used in RT-qPCR assay

	Genes	Primer sequences (5′–3′)
Cattle	β-actin F	CCAACCGTGAGAAGATGAC
	β-actin R	AGGCATACAGGGACAGCACA
	IL-1β F	AAAAATCCCTGGTGCTGGCT
	IL-1β R	CATGCAGAACACCACTTCTCG
	IL-6 F	CACTGACCTGCTGGAGAAGATGC
	IL-6 R	CCGAATAGCTCTCAGGCTGAACTG
	TNF-α F	GCTGACGGGCTTTACCTCATCTAC
	TNF-α R	GGCTCTTGATGGCAGACAGGATG
	Nrf2 F	GTCGTCGGGGAGCCTCAAAG
	Nrf2 R	AATCCATGTCCTGCTGGGACG

RT-qPCR, reverse transcription quantitative polymerase chain reaction.

**Table 2:** The primer sequences (5′–3′) of mouse used in RT-qPCR assay

	Genes	Primer sequences (5′–3′)
Mouse	β-actin F	GGCTGTATTCCCCTCCATCG
	β-actin R	CCAGTTGGTAACAATGCCATGT
	IL-1β F	AACCTGCTGGTGTGTGACGTTC
	IL-1β R	CAGCACGAGGCTTTTTTGTTGT
	IL-6 F	GACAAAGCCAGAGTCCTTCAGA
	IL-6 R	TGTGACTCCAGCTTATCTCTTGG
	TNF-α F	CGTCAGCCGATTTGCTATCT
	TNF-α R	CGGACTCCGCAAAGTCTAAG
	Nrf2 F	TCTTGGAGTAAGTCGAGAAGTGT
	Nrf2 R	GTTGAAACTGAGCGAAAAAGGC

RT-qPCR, reverse transcription quantitative polymerase chain reaction.

**Table 3:** The primer sequences (5′–3′) of miRNA used in RT-qPCR assay

Genes	Primer sequences (5′–3′)
U6 RT	AACGCTTCACGAATTTGCGT
U6 F	CTCGCTTCGGCAGCACA
U6 R	AACGCTTCACGAATTTGCGT
miR-27b RT	GTCGTATCCAGTGCAGGGTCCGAGGTATTCGC ACTGGATACGACGCAGAA
miR-27b F	CTTCACAGTGGCTAAGTTCTGC
miR-27b R	ATCCAGTGCAGGGTCCGAGG

miRNA, microRNA; RT-qPCR, reverse transcription quantitative polymerase chain reaction.

## Data Availability

Upon reasonable request, the datasets of this study can be available from the corresponding author.

## SUPPLEMENTARY MATERIAL

**Supplement 1**. GO functional enrichment analysis. **Supplement 2**. KEGG pathway enrichment analysis.
