# Supplementary material for: miR-27b-3p aggravates steatohepatitis progression by targeting the Nrf2/HO-1 pathway
Source: Anim Biosci. 2026 Jul 27;39(11):260476. doi: 10.5713/ab.260476 (PMC13740664; doi:10.5713/ab.260476)

## Supplement 1. GO functional enrichment analysis

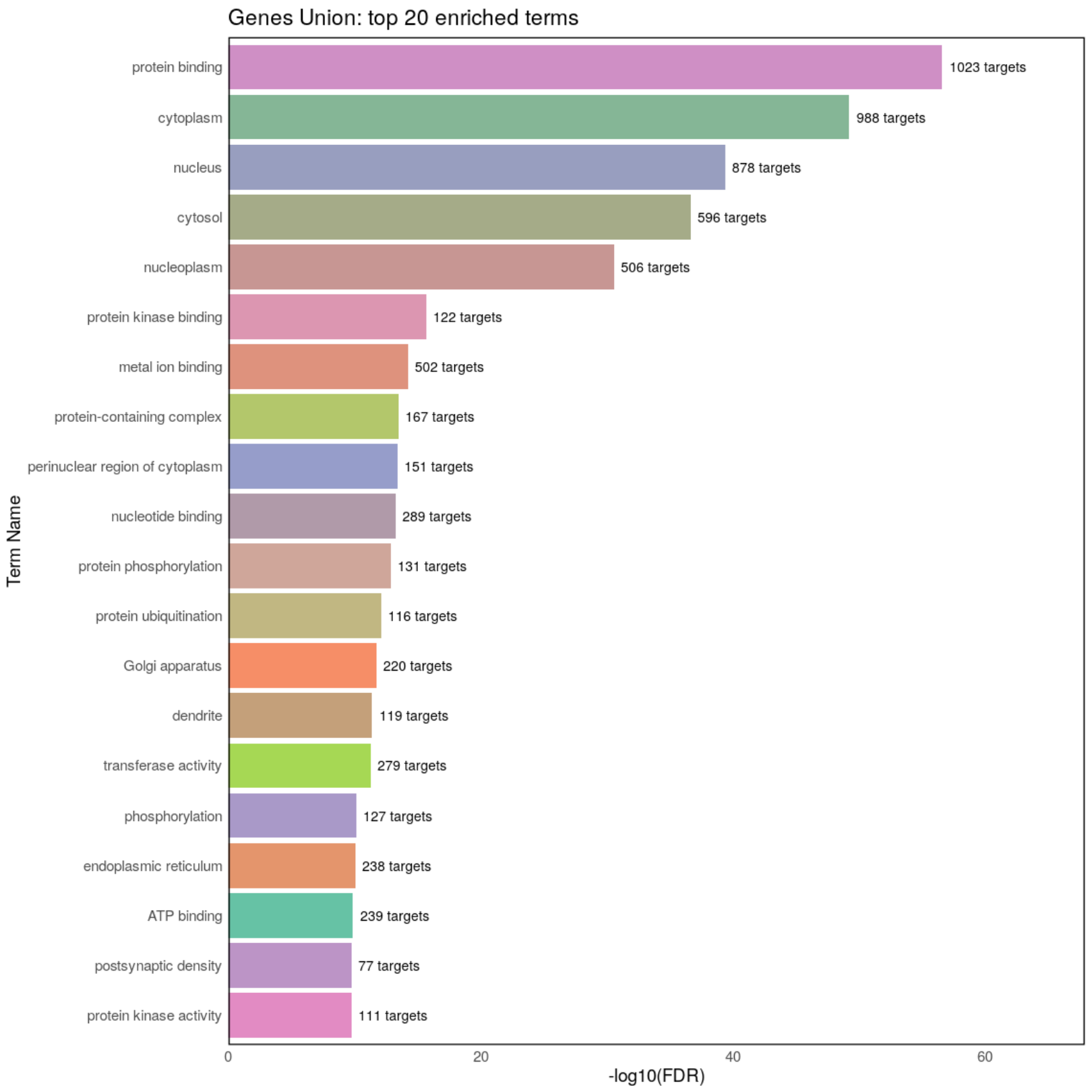


## Supplement 2. KEGG pathway enrichment analysis

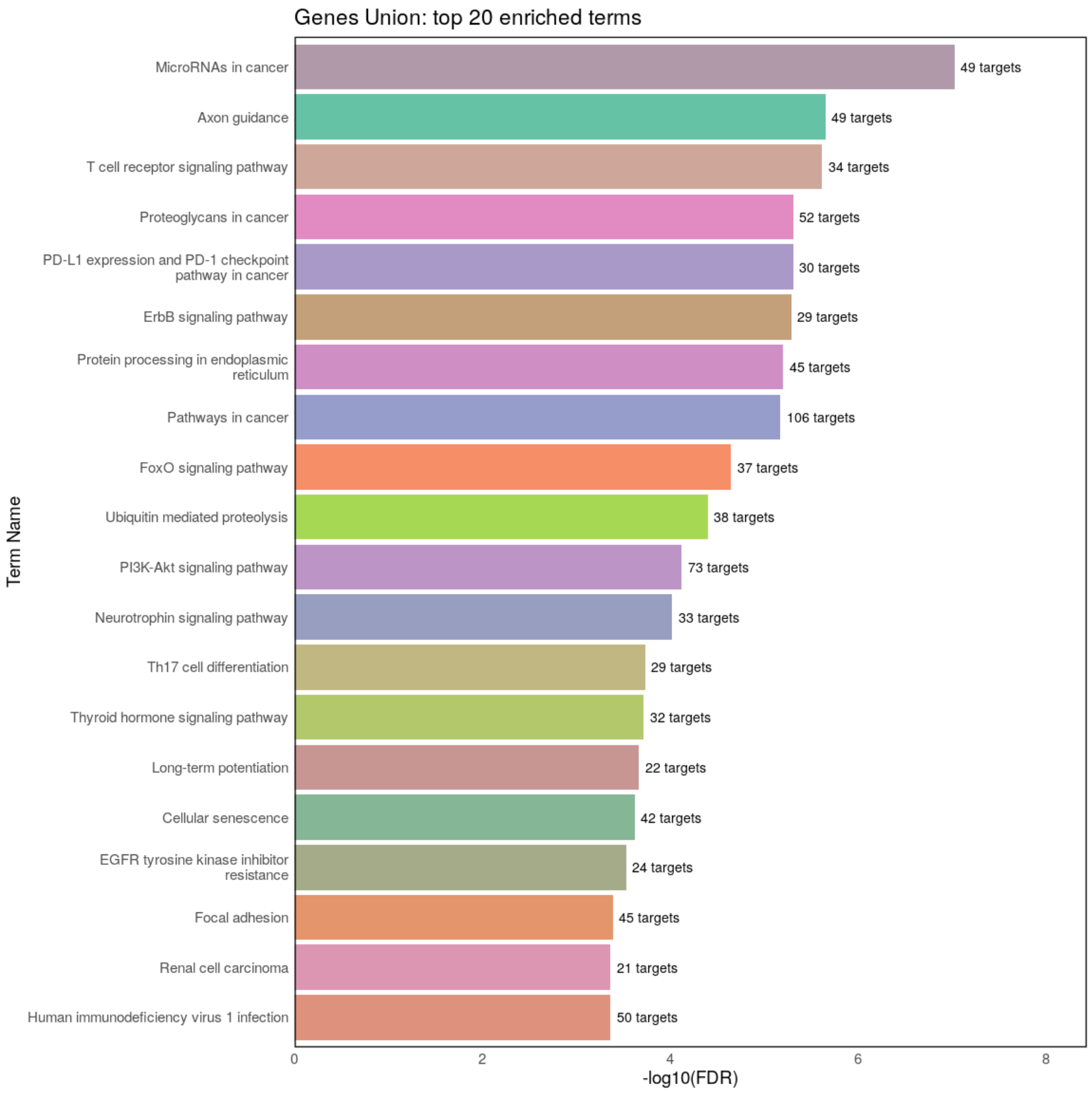

Supplement: Supplementary file 1 [file ab-260476-Supplementary.pdf]
